# Left Ventricular Systolic Function in Asymptomatic Men Aged 65–75 Years, Relation to Insulin Resistance and Pre-Diabetes: A DANCAVAS Cross-Sectional Sub-Study

**DOI:** 10.3390/jcdd12050180

**Published:** 2025-05-13

**Authors:** Johanna Larsson, Søren Auscher, Freja Sønder Madsen, Katrine Schultz Overgaard, Gokulan Pararajasingam, Laurits Juhl Heinsen, Thomas Rueskov Andersen, Jes Sanddal Lindholt, Jess Lambrechtsen, Kenneth Egstrup

**Affiliations:** 1 Cardiovascular Research Unit, Odense University Hospital Svendborg, Baagøes Allé 15, 5700 Svendborg, Denmarkthomas.rueskov.andersen@rsyd.dk (T.R.A.); 2 Department of Cardiology, Odense University Hospital Svendborg, Baagøes Allé 15, 5700 Svendborg, Denmark; soeren.auscher@rsyd.dk (S.A.);; 3 Department of Cardiac, Thoracic and Vascular Surgery, Odense University Hospital Odense, J.B. Winsløws Vej 4, 5000 Odense, Denmark; jes.sanddal.lindholt@rsyd.dk

**Keywords:** asymptomatic, insulin resistance, pre-diabetes, global longitudinal strain, diastolic dysfunction

## Abstract

Aim: Insulin resistance (IR) and hyperglycemia have been associated with increased risk of heart failure (HF) in patients with and without diabetes. Global longitudinel strain (GLS) has been shown to be superior in the detection of left ventricular (LV) systolic dysfunction when compared to ejection fraction (EF). In this study, we aimed to assess GLS in relation to IR and pre-diabetes. Method: All participants underwent an echocardiography to assess LV systolic function using GLS. IR was evaluated using homeostatic model assessment for IR (HOMA-IR), and the participants were divided into tertiles based on their HOMA-IR values. An oral glucose tolerance test (OGTT) was performed to divide participants into normal glucose tolerance (NGT) and pre-diabetes. A multivariable linear regression model was used to assess GLS in relation to IR and glycemic groups. Results: In total, 359 men without significant coronary artery disease (CAD) and without diabetes were enrolled. Participants in the higher HOMA-IR tertile had significantly reduced GLS when compared with participants in the lower HOMA-IR tertile (−17.9% vs. −18.7%, *p* < 0.01). A significant trend was observed towards reduced GLS with increasing HOMA-IR tertile (*p*-trend 0.005). However, in the multivariable regression model, only waist-to-height-ratio (WH) (β 7.1 [95% CI 3.1–11.1, *p* = 0.001) remained significantly associated with GLS, whereas HOMA-IR tertile and pre-diabetes were not. Conclusions: In asymptomatic elderly men with no diabetes or CAD, neither IR nor pre-diabetes was associated with GLS in the adjusted regression model. Increased WH seems to be associated with reduced systolic function by GLS measurement.

## 1. Introduction

Diabetes mellitus (DM) is associated with a two- to five-times increased risk of heart failure (HF) compared to individuals without diabetes [1,2]. The increased risk is not solely explained by the frequent coexistence of cardiovascular disease (CVD) and hypertension. Hyperglycemia and hyperinsulinemia seem to be associated with HF in individuals both with and without diabetes [3,4,5], implying that structural changes in the left ventricle (LV) may occur before the onset of manifest type 2 DM (T2DM). Accordingly, pre-diabetes [4,6] and insulin resistance (IR) [7,8] have previously been found to be associated with HF in some, but not all, studies [9].

Global longitudinal strain (GLS) measures the deformation of the myocardial tissue during a cardiac cycle [10], and it is considered to be more sensitive to subtle changes in LV systolic function compared to LV ejection fraction (LVEF) [11]. In previous studies, GLS has been reported to predict significant coronary artery disease (CAD) in patients with suspected stable angina pectoris (SAP) [12], and to predict cardiovascular events and all-cause mortality [13,14]. Several studies have examined GLS in relation to IR and pre-diabetes [15,16,17,18,19,20], and most of them reported subtle reductions in GLS with increasing IR and in patients with pre-diabetes compared to individuals with normal glucose tolerance (NGT). However, most of these studies did not perform a direct test, e.g., coronary computed tomography (CCTA) or coronary angiography (CAG), to exclude participants with significant coronary artery stenosis, which is known to be a confounder for impaired GLS. Studies assessing GLS in participants with no significant coronary artery stenosis are lacking. Therefore, we aimed to assess GLS in relation to IR and glycemic groups in asymptomatic men aged 65–75 years without known CAD or diabetes.

## 2. Methods

### 2.1. Study Design and Study Poulation

**Figure 1:**
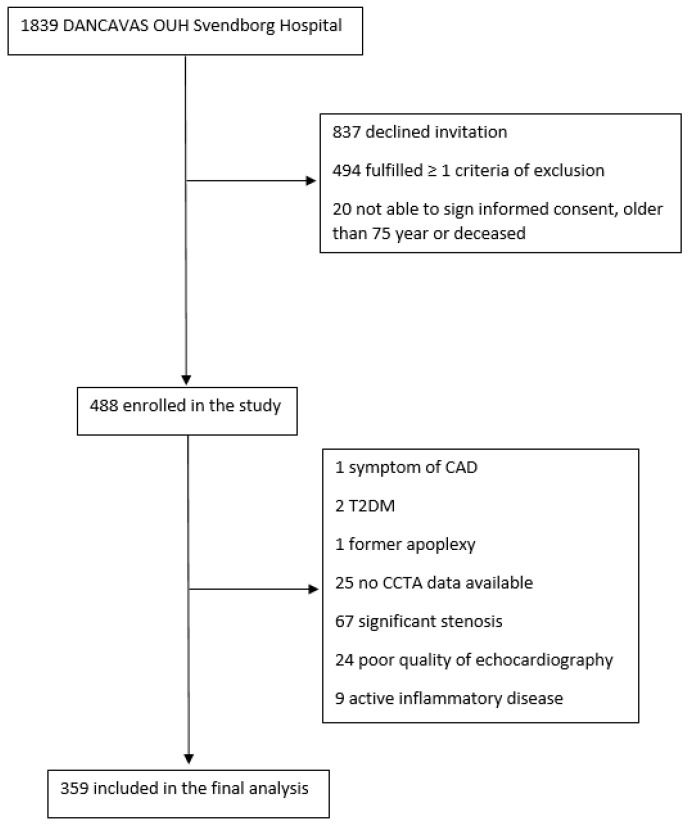
Flowchart of recruitment for study participants. Flowchart for available participants reaching the final study population. CAD = coronary artery disease; T2DM = type 2 diabetes mellitus; CCTA = coronary computed tomography angiography.

This descriptive, cross-sectional, single-center study was performed at the Odense University Hospital (OUH) Svendborg Hospital, Denmark, from May 2016 to July 2019. The study participants were recruited from the large randomized multicenter population-based Danish Cardiovascular Screening (DANCAVAS) trial [21,22]. The original study population has previously been described [23]. It consisted of 450 participants recruited to evaluate the association between vulnerable plaque composition, high-risk plaque occurrence, and GLS in relation to IR and pre-diabetes in a population of asymptomatic men with no diabetes. In the original population, we included male participants from the DANCAVAS study, who had an unenhanced CCTA at OUH Svendborg Hospital, were aged 65–75 years, and were without known T2DM, CAD, or stroke. Furthermore, participants with symptoms of CAD (New York Heart Association classification > II or typical angina), estimated glomerular filtration rate (eGFR) < 45 mL/min, iodine contrast allergy, untreated hyperthyriodism, or active inflammatory disease were excluded. In this study assessing GLS, we only included participants without significant coronary stenosis. Therefore, all participants underwent a contrast-enhanced CCTA (GE Revolution CT, GE Healthcare, Waukesha, WI, USA), and the coronary arteries were analyzed using the validated semi-automated software system (QAngioCT Research Edition v.3.1.3.16, Medis Medical Imaging Systems, Leiden, The Netherlands) [24]. Participants with either a non-diagnostic CCTA scan or a significant stenosis defined as a >50% diameter stenosis of left anterior descending (LAD), left circumflex (LCX), or right coronary artery (RCA) were excluded. Finally, we excluded participants with a non-diagnostic echocardiography, moderate to severe valve stenosis or insufficiency, atrial fibrillation, or other arrhythmia interfering with our analysis (Figure 1).

### 2.2. Echocardiography

Echocardiography was performed using a Vivid-9 E90 or a Vivid E95 (GE Healthcare, Chicago, IL, USA). All images were stored digitally and pseudonymized to allow the blinding of patient data. All analyses were performed offline using EchoPac software version 202, revision 50 (GE Healthcare). Intra- and inter-observer variability were assessed in 20 participants. All observers were blinded to all clinical data.

LVEF was assessed using Simpson’s biplane method [25]. Left ventricle mass (LVM) was evaluated using the Devereux formula (0.8 × 1.04 × (left ventricle end-diastolic dimension + posterior wall end-diastolic dimension + interventricular septum end-diastolic dimension)^3 − (left ventricular end-diastolic dimension)^3) + 0.6 g [26]. Thereafter, LVM was divided by body surface area (BSA) using the Dubois formula (0.00718 × (height [cm]^0.725) × (weight [kg]^0.425)) to receive the indexed LVM (LVMI). Left atrial volume was also indexed by BSA. All diastolic measurements were averaged for three consecutive heart beats, and an apical four-chamber view was used. Pulsed-wave (PW) Doppler through the tip of the mitral leaflet was used to measure the inflow in the left ventricle. The peak early (E) and late (A) diastolic filling velocity, as well as the deceleration time of E (DCT), were measured. PW tissue Doppler was placed within 1 cm of the mitral annulus at septal and lateral positions, and peak early diastolic mitral annular tissue velocity (e’) was collected. Septal and lateral e’ were thereafter averaged and used in E/e’.

### 2.3. Global Longitudinal Strain

We assessed GLS using the Q-analysis function in the EchoPac software version 202, revision 50 (GE Healthcare). Strain analysis was performed on two-dimensional (2D) images of the apical four-chamber, two-chamber, and long-axis views with an optimized frame rate between 58 and 80 frames/s. Aortic valve closure (AVC) and aortic valve opening (AVO) were set manually using continuous wave (CW) through the aortic valve. GLS was obtained after a satisfactory tracing of speckles of the endocardium.

### 2.4. Insulin Resistance, Oral Glucose Tolerance Test, and Blood Sample

Insulin resistance was assessed using the homeostatic model assessment for IR (HOMA-IR), according to the calculation HOMA-IR = [fasting insulin concentration (µIU/ml) × fasting plasma glucose concentration (mmol/L)]/22.5 [27]. The originally included study population consisting of 450 participants was divided into tertiles according to the HOMA-IR: lower HOMA-IR tertile (L-IR) (<1.93), middle HOMA-IR tertile (M-IR) (1.93–3.1), and higher HOMA-IR tertile (H-IR) (>3.1). We also assessed IR using the triglyceride glucose (TyG) index according to the following calculation: Ln (fasting triglycerides concentration (mg/dL) × fasting plasma glucose concentration (mg/dL)/2) [28]. All participants underwent one standard oral glucose tolerance test (OGTT) after a minimum of 10 h fasting. The OGTT was performed according to the World Health Organization (WHO) recommendations [29]. In the present study, we defined normal glucose tolerance (NGT) as fasting plasma glucose (FPG) < 6.1 and 2 h plasma glucose (2HPG) < 7.8 mmol/L, and pre-diabetes as FPG ≥ 6.1 or 2HPG ≥ 7.8 mmol/L. Furthermore, the participants’ biochemical parameters were analyzed for glycated hemoglobin A1c (HbA1c), fasting lipid parameters (total cholesterols, low-density lipoprotein (LDL), high-density lipoprotein (HDL), and triglycerides), creatinine, and high-sensitivity C-reactive protein (hs-CRP).

### 2.5. Data Collection

Demographic data, medical history, and medication use were collected by interviews and reviews of patient files. We defined hypertension and hypercholesterolemia as the use of at least one antihypertensive medication and the use of lipid-lowering medication, respectively. Smoking status was categorized as never, former or active smoker. Lifetime exposure to tobacco was calculated in pack-years (one pack-year = 20 cigarettes (=16 g tobacco) daily for 1 year). Family predisposition was defined according to guidelines [30]. BMI was calculated as weight (kilogram (kg))/height^2^ (metres (m)^2^). Waist was measured horizontally midway between the most distal costae and the top of the iliac crest, and hip was measured horizontally at the broadest point of the buttocks. Waist-to-height-ratio (WH) was calculated as waist divided by height. Blood pressure was measured twice after a minimum of 15 min rest.

### 2.6. Statistics

All statistical analyses were performed using STATA IC 17 (StataCorp, College Station, TX, USA). Continuous variables with visual normal distribution were reported as means and standard deviations (SD) and variables with visual non-normal distribution as medians and interquartile ranges (IQR). Categorical variables were reported as numbers (*n*) and percentages (%). Unpaired Student’s *t*-test was used to assess differences between continuous values with normal distribution, whereas the Mann–Whitney test was used to assess variables with non-normal distribution. Fisher’s least significant difference procedure was used to assess differences between three groups [31]. The Chi-Square test was used to assess differences between categorical values, and Fisher’s exact test was used to assess differences between three groups. Linear regression was used for the analysis of GLS in relation to HOMA-IR and glycemic groups. The influence of possible confounders was assessed using the multivariable regression model and included age, WH, hypertension, smoking exposure, statin use, systolic blood pressure, and HbA1c. In the final multivariable regression models, we included variables with *p* < 0.05 from the univariable models. The variance inflation factor (VIF) was used to test for multicollinearity in the final model, where VIF > 5 indicates that multicollinearity may be present. To assess trends between GLS and HOMA-IR tertiles and glycemic groups, we used Cuzick’s test. Bland–Altman’s 95% limits of agreement (LOA) and Pearson’s correlation coefficient (r) were used to assess the reproducibility of GLS [32].

A two-sided *p* < 0.05 was considered as statistically significant.

### 2.7. Ethics

This study was performed in accordance with the revised Helsinki Declaration regarding ethical principles for medical research involving human subjects. All participants received both written and oral information before informed consent was signed. The study was approved by the ethics committee of the Region of Southern Denmark, project ID (S-20160024), and by the Danish Data Protection Agency, project ID (16/6574). The trial was registered at Clinical.Trial.gov, project ID NCT04525508. Research Electronic Data Capture (REDCAP) was used for secure data storage.

## 3. Results

### 3.1. Clinical Characteristics

In total, 359 men with a mean age of 70 ± 3 years were included (Table 1). Hypertension was treated in 116 participants (32%). Furthermore, mean systolic and diastolic blood pressure was 138 ± 18 and 81 ± 10 mmHg, respectively. Pre-diabetes was identified in 165 (46%) participants, and median HOMA-IR was 2.4 IQR [1.7; 3.6].

Demographics, use of medication, and measurements according to HOMA-IR tertiles and glycemic groups are displayed in Supplementary Files S1 and S2. Participants in the H-IR had significantly different glucometabolic parameters, BMI, waist circumference, WH, hip measurement, triglycerides, HDL, hs-CRP, systolic and diastolic blood pressure compared with the participants in the L-IR. Furthermore, significantly more participants in the H-IR had hypertension compared with participants in the L-IR (38% vs. 23%).

Participants with pre-diabetes showed significant differences in glucometabolic measurements, BMI, waist circumference, WH, hip measurement, HDL, triglycerides and hs-CRP, as well as systolic and diastolic blood pressure compared to participants with NGT (Supplementary File S2). Additionally, participants with pre-diabetes had significantly greater smoking exposure measured in pack-years (20.3 ± 21.0 vs. 12.2 ± 15.9 years), and had a significantly higher frequency (39% vs. 26%) compared to participants with NGT.

### 3.2. Echocardiography

**Figure 2:**
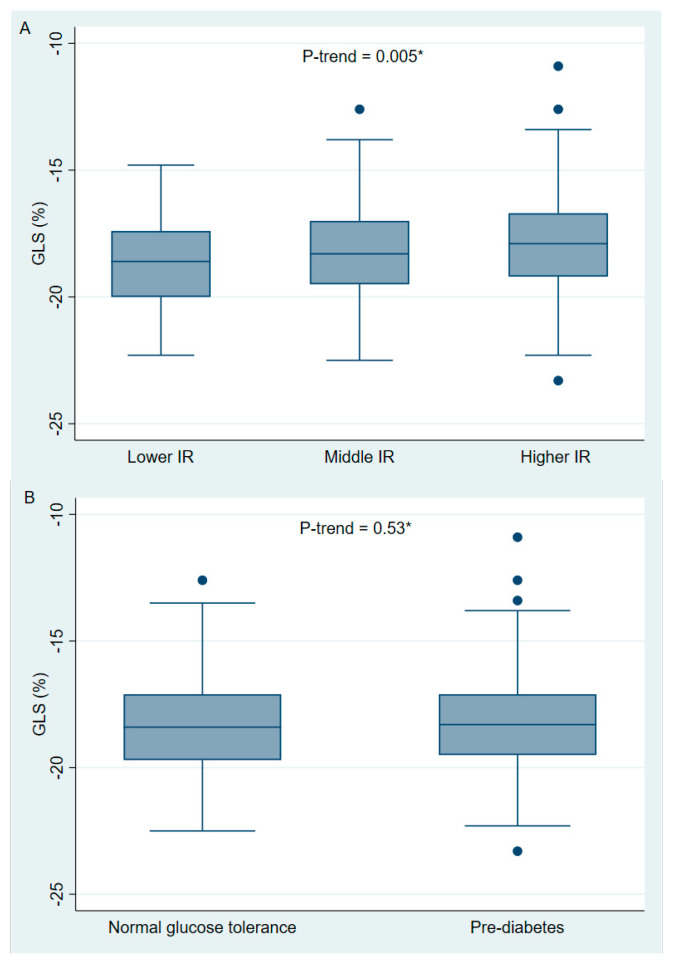
Boxplot of GLS in relation to HOMA-IR tertiles and glycemic groups in 359 participants. (**A**) GLS according to the HOMA-IR tertiles. (**B**) GLS according to glycemic status. GLS = global longitudinal strain; HOMA-IR = homeostatic model assessment for IR, * Cuzick’s test for trend between the groups.

Overall, mean GLS was −18.3 ± 1.9% and mean LVEF was 61.9 ± 5.2% (Table 2). The participants in the H-IR had reduced GLS compared with the participants in the L-IR (−17.9 ± 2.1 vs. −18.7% ± 1.7, *p* < 0.01), and a significant trend was observed for GLS across the HOMA-IR tertiles (*p*-trend = 0.005) (Figure 2). There was no significant difference in LVEF when the participants in the H-IR were compared with the participant in the L-IR (61.7 ± 5.1% vs. 62.2 ± 5.3, overall *p* = 0.75). Furthermore, E/A was lower in the participants in the H-IR compared with the participants in the L-IR (0.85 IQR [0.76; 1] vs. 0.94 IQR [0.8; 1.06], *p* < 0.01), whereas LAVI, LVMI, and E/e’ did not differ significantly between the groups.

In participants with pre-diabetes, neither GLS (−18.2 ± 2.0 vs. −18.3 ± 1.8%, *p* = 0.42) nor LVEF (61.5 ± 5.3 vs. 62.3 ± 5.0%, *p* = 0.15) differed significantly when compared to participants with NGT (Table 3). Moreover, we did not find any significant trend between GLS and glycemic groups (*p*-trend = 0.53) (Figure 2). Finally, E/e’ was higher in participants with pre-diabetes compared to participants with NGT (8.31 IQR [7.13; 10.50] vs. 8.07 [6.75; 9.46], *p* = 0.03), whereas E/A, LVMI and LAVI did not differ significantly between the two groups.

### 3.3. Uni- and Multivariable Regression Analysis

In the univariable model, GLS was significantly associated with the M-IR and H-IR tertiles, systolic blood pressure, and WH, whereas pre-diabetes, statin use, hypertension, pack-years, age, and HbA1c were not (Table 4). In the multivariable linear regression model, only WH remained significantly associated with GLS (β 7.1 [95% CI 3.1–11.1], *p* = 0.001). The final regression model was also tested for the presence of multicollinearity, where all VIF values were <2.1 indicating no major influence of multicollinearity. We also assessed IR using TyG in univariable and multivariable regression models (Supplementary File S3). In the univariable regression model, TyG was significantly associated with GLS, but this did not remain significant in the multivariable regression model, where only WH remained significantly associated with GLS.

### 3.4. Intra- and Inter-Observer Variability

The reproducibility of GLS data showed good agreement with Pearson’s correlations coefficient of 0.80 and 0.86 for inter- and intra-observer variability, respectively.

## 4. Discussion

In this study, we investigated GLS in relation to IR and pre-diabetes in asymptomatic men aged 65–75 years without known CAD or diabetes. The main findings in this study were as follows: (1) There was an univariable trend towards a subtle reduction in GLS with increasing HOMA-IR tertile, but this did not remain significant in the adjusted models. (2) Pre-diabetes was not associated with GLS. (3) In the multiple regression model, only WH remained significantly associated with GLS.

### 4.1. Insulin Resistance and Heart Failure

Assessment of GLS is reported to better identify subtle reductions in the LV systolic function than LVEF [11]. The Copenhagen City Heart study assessed 1296 persons from the general population and reported an increased risk of the composite endpoint (HF, acute myocardial infarction (AMI), and cardiovascular death) with lower GLS [33]. Furthermore, they concluded that GLS was better than both the Framingham Risk Score and the SCORE risk chart in predicting the mentioned composite endpoint. In the present study, we observed a significant trend toward a subtle reduction in GLS with increasing HOMA-IR tertile, but the trend did not remain significant in the adjusted model. We also assessed IR using TyG index in relation to GLS in the linear regression model, but found no significant association in the adjusted model. Previous studies have assessed IR in relation to GLS [15,16,17,18,20], and most of the studies reported significant associations between GLS and IR. However, there are important differences between the present study and the previous studies. In the present study, all participants underwent a CCTA to assess coronary artery stenosis. Impaired GLS predicted significant coronary artery stenosis in patients with suspected stable angina pectoris, and is therefore a potential confounder when assessing GLS [12]. Furthermore, the extent of CAD has been associated with GLS among individuals with preserved LVEF [34]. In comparison, most previously published studies did not systematically assess stenosis in all included participants. However, in a small study by Atici et al. [17], they included 118 participants with normal myocardial perfusion, but they did not assess the anatomy of the coronary arteries. Furthermore, the present study sought to assess GLS in participants aged 65–75 years. Only one study by Garg et al. [15] had a comparable study population with a mean age > 60 years. They reported a significant association between longitudinal strain and HOMA-IR, Matsuda insulin sensitivity index (ISI), FPG, and 2HPG, but after adjusting for waist circumference, neither HOMA-IR, FPG nor 2HPG remained significantly associated with GLS. These findings are in agreement with our current findings, where HOMA-IR was no longer associated with GLS after including WH in the regression model. Previously, Vardenly et al. [8] reported a strong association between IR and the incidence of HF among younger individuals. The combination of age-mediated changes in the LV and the increased prevalence of comorbidities, e.g., hypertension and/or increasing BMI with increasing age, may diminish the effect of IR on GLS. Therefore, larger, preferably longitudinal studies are wanted to further assess the effect of IR on GLS.

### 4.2. Pre-Diabetes and Heart Failure

The association between pre-diabetes and GLS has previously been assessed in a limited number of studies [18,19]. They seem to indicate reduced GLS in participants with pre-diabetes compared to participants with NGT. However, we did not find such an association in the present study. Several important differences are to be noted though, when comparing the present study with previously published studies. As mentioned above, significant stenosis is an important confounder, which has not been assessed systematically in the previously published studies. Furthermore, the mentioned studies used the American Diabetes Association’s (ADA) definition of pre-diabetes [18,19] as including HbA1c, which was more significant according to the univariable regression models in our study. Finally, our population consisted of participants aged 65–75 years, and only Skali et al. [19] had a similar study population with a mean age >60 years. They reported a significant association between pre-diabetes and GLS, although the reduction was subtle (mean adjusted GLS −18.2% vs. −18.4% for pre-diabetes and NGT, respectively), in a study population of 4419 participants. Our study adds information regarding the association between pre-diabetes and GLS in men aged 65–75 years with no significant stenosis in the coronary arteries. The heterogenicity between the previous studies highlights the need for further studies to explore the matter.

### 4.3. Obesity and Heart Failure

In the multivariable regression model, the association between GLS and HOMA-IR was no longer significant when WH was included. Obesity has previously been associated with an increased risk of HF [35] and with reduced GLS [20,36]. Furthermore, a more recent study has reported increased hospitalization for HF with increasing BMI and WH in patients with HF with reduced EF (HFrEF), where WH seemed to be better at predicting the selected outcome than BMI [37]. In relation to HF, obesity has been linked to increased free fatty acids [38] and increased oxidative stress/inflammation [39,40]. These processes may lead to increased apoptosis [38] as well as fibrosis [41] and lipid deposition in the myocardial tissue [42], which ultimately cause stiffness and thus the impairment of the ventricular function [41]. Furthermore, obesity may affect LV systolic function by contributing to increased arterial stiffness [43,44,45], thereby increasing afterload, or by increasing the cardiac load through an increase in blood volume [46]. Finally, central obesity may affect LV systolic function, as increasing WH is associated with both a higher risk of ischemic CVD [47] and cardiovascular risk factors such as hypertension [48]. Insulin resistance and obesity, especially central obesity, are closely related [49]. Accordingly, BMI, WH, and waist circumference all increased significantly across the HOMA-IR tertiles. Similarly, these parameters were elevated in participants with pre-diabetes participants compared to those with NGT. Still, HOMA-IR was not associated with GLS in the adjusted model.

### 4.4. Limitations

Firstly, we included solely men aged 65–75 years, and thus our results may not apply in younger persons or in women. Secondly, since this study is a relatively small cross-sectional study, bias cannot be excluded due to the study design. Thirdly, although all participants with known T2DM were excluded from this study, 42 participants had an OGTT within the diabetic threshold (FPG ≥ 7 and/or 2HPG ≥ 11.1). We categorized these participants as having pre-diabetes, as they did not fully meet the diagnostic criteria for diabetes and showed no symptoms of diabetes, and the cross-sectional design did not allow for repeated measurements. This categorization may have affected the results; however, it is less likely, as neither pre-diabetes nor HOMA-IR tertiles were associated with GLS in the adjusted models. Fourthly, we assessed HOMA-IR in relation to GLS. Several methods exist for evaluating IR. HOMA-IR, which is based on fasting insulin concentration, primarily reflects hepatic IR. The TyG index, a more easy accessible method, uses fasting triglyceride and FPG concentrations. Both HOMA-IR and TyG index have been validated against the hyperinsulinemic–euglycemic clamp [27,50]. However, repeated measurements of insulin concentrations during the OGTT would have been preferable to assess peripheral IR in addition to the hepatic IR. Finally, WH was associated with GLS in the multivariable analysis. Increased central adiposity often lowers the quality of the echocardiography, and thus may have led to reduced GLS, although participants with reduced quality of echocardiography were excluded in the analysis.

Clinical implications: The prevalence of obesity is rapidly increasing, and consequently, the number of individuals with IR and pre-diabetes is also increasing. In this study, the adjusted analysis did not demonstrate significant association between LV systolic function and either IR or pre-diabetes. However, our findings indicate that central obesity is associated with reduced LV systolic function, independent of traditional cardiovascular risk factors. Given the close relationship between central obesity, IR, and pre-diabetes, this association need to be further investigated. The observed significant association between central obesity and impaired LV systolic function indicates the need for continued efforts to inform overweight individuals about the potential adverse impact of central obesity on LV systolic function, and to encourage weight reduction as well as lifestyles changes. The results also suggest that WH, as a measure of central obesity, may be a useful tool for use in everyday clinical practice to evaluate individuals at risk of HF, although this should be further validated in larger trials.

## 5. Conclusions

Among men aged 65–75 years without known diabetes and without significant CAD, we found an unadjusted significant trend towards reduced systolic function, assessed by GLS, with increasing HOMA-IR tertiles. However, in the adjusted model, neither HOMA-IR nor pre-diabetes were associated with GLS, and only WH remained significantly associated with GLS.

## Figures and Tables

**Table 1:** Demography, medication and measurements in 359 participants.

	All (*n* = 359)
Age, years	70 ± 3
Hypertension, *n* (%)	116 (32)
Hypercholesterolemia, *n* (%)	120 (33)
Smoking, *n* (%)	
Active	39 (11)
Previous	185 (52)
Never	132 (37)
Pack-years	15.9 ± 18.8
Aspirin, *n* (%)	95 (26)
ACEIs/ARBs, *n* (%)	92 (26)
Beta-blocker, *n* (%)	22 (6)
Statins, *n* (%)	119 (33)
Duration statin treatment, months, median [IQR]	17 [3; 60]
Systolic blood pressure, mmHg	138 ± 18
Diastolic blood pressure, mmHg	81 ± 10
BMI, kg/m^2^	27 ± 3
Waist circumference, cm	99 ± 10
Hip, cm	103 ± 6
Waist-to-height-ratio	0.56 ± 0.06
HbA1c, mmol/mol	36.8 ± 3.7
HDL, mmol/L	1.6 ± 0.4
LDL, mmol/L	2.8 ± 1.0
Total cholesterols, mmol/L	4.9 ± 1.0
Triglycerides, mmol/L	1.1 ± 0.6
Prediabetes, *n* (%)	165 (46)
HOMA-IR, * median [IQR]	2.4 [1.7; 3.6]
FPG, mmol/L	5.9 ± 0.6
2HPG, mmol/L	7.0 ± 2.2
TyG index	8.4 ± 0.48
hs-CRP, mg/L	2.1 ± 2.6

Continuous values are presented as mean ± standard deviation (SD) or median and [inter quartile range], and categorical data as number (*n*) and percentage (%). BMI = body mass index, ACEI = angiotensin-converting enzyme inhibitors; ARB = angiotensin receptor blockers; HbA1c = hemoglobin A 1c; HDL = high-density lipoprotein; LDL = low-density lipoprotein; HOMA-IR = homeostatic model assessment for IR; FPG = fasting plasma glucose; 2HPG = two-hour plasma glucose; hs-CRP = high-sensitivity C-reactive protein; TyG index = triglyceride glucose index. * HOMA-IR available for 358 participants.

**Table 2:** Echocardiography according to HOMA-IR tertiles in 359 participants *.

	All (*n* = 359)	Lower HOMA-IR Tertile (*n* = 119)	Middle HOMA-IR Tertile (*n* = 124)	Higher HOMA-IR Tertile (*n*= 115)	*p*-Value
GLS (%)	−18.3 ± 1.9	−18.7 ± 1.7	−18.2 ± 1.8	−17.9 ± 2.1 **	**0.02**
LVEF (%) †	61.9 ± 5.2	62.2 ± 5.3	61.7 ± 5.2	61.7 ± 5.1	0.75
LAVI (mL/m^2^) ††	24.6 ± 7.8	25.2 ± 8.8	24.7 ± 7.2	23.6 ± 6.9	0.42
LVMI (g/m^2^) †††	65.1 ± 14.8	64.9 ± 14.7	65.5 ± 15.5	64.9 ± 14.2	0.87
E/e’ [IQR]	8.24 [6.86; 9.85]	8 [6.57; 9.23]	8.17 [7.03; 9.82]	8.40 [7.38; 10.46]	0.08
E/A [IQR]	0.89 [0.78; 1.02]	0.94 [0.8; 1.06]	0.87 [0.75; 1.02]	0.85 [0.76; 1] **	**0.01**

Continuous values are presented as mean ± standard deviation (SD) or as median and [inter quartile range]. Categorical data are presented as number (*n*) and percentage (%). LVEF = left ventricular ejection fraction; GLS = global longitudinal strain; LAVI = left atrial volume index; LVMI = left ventricular mass index; E/e’ = ratio of peak early diastolic filling velocity (E) and average peak early diastolic mitral annular tissue velocity (e’); E/A = ratio of peak early diastolic filling velocity (E) and peak late diastolic filling velocity (A); HOMA-IR = homeostatic model assessment for IR; IQR = interquartile range. * HOMA-IR available for 358 participants. ** Higher HOMA IR vs. lower HOMA-IR, *p* < 0.01. † LVEF available in 356 participants. †† LAVI available in 346 participants. ††† LVMI available in 354 participants.

**Table 3:** Echocardiography according to glycemic status in 359 participants.

	Normal Glucose Tolerance (*n* = 194)	Pre-Diabetes (*n* = 165)	*p*-Value
GLS (%)	−18.3 ± 1.8	−18.2 ± 2.0	0.42
LVEF (%) †	62.3 ± 5.0	61.5 ± 5.3	0.15
LAVI (mL/m^2^) ††	24.0 ± 7.8	25.2 ± 7.8	0.15
LVMI (g/m^2^) †††	64.0 ± 14.1	66.4 ± 15.4	0.13
E/e’ [IQR]	8.07 [6.75; 9.46]	8.31 [7.13; 10.50]	**0.03**
E/A [IQR]	0.91 [0.78; 1.04]	0.87 [0.77; 1.01]	0.11

Continuous values are presented as mean ± standard deviation (SD) or as median and [inter quartile range], and categorical data as number (*n*) and percentage (%). LVEF = left ventricular ejection fraction; GLS = global longitudinal strain; LAVI = left atrial volume index; LVMI = left ventricular mass index; E/e’ ratio of peak early diastolic filling velocity (E) and average peak early diastolic mitral annular tissue velocity (e’); E/A = ratio of peak early diastolic filling velocity (E) and peak late diastolic filling velocity (A); HOMA-IR = homeostatic model assessment for IR; IQR = interquartile range. † LVEF available in 357 participants. †† LAVI available in 347 participants. ††† LVMI available in 355 participants.

**Table 4:** Uni- and multivariable linear regression models for 359 participants.

	Univariable			Multivariable *		
	β	95% CI	*p*-Value	β	95% CI	*p*-Value
GLS						
Pre-diabetes	0.16	−0.24–0.56	0.42			
HOMA IR tertile						
Lower	ref.					
Middle	0.48	0.005–0.96	**0.048**	0.09	−0.42–0.61	0.73
Higher	0.74	0.26–1.2	**0.003**	0.11	−0.47–0.69	0.72
Statin use	0.26	−0.16–0.68	0.23			
Hypertension	0.37	−0.06–0.79	0.09			
Systolic blood pressure, mmHg	0.01	0.003–0.03	**0.01**	0.01	−0.0002–0.02	0.054
Pack-years	−0.002	−0.01–0.008	0.67			
WH	7.9	4.6–11.3	**<0.001**	7.1	3.1–11.1	**0.001**
Age	0.0008	−0.07–0.07	0.98			
HbA1c	0.05	−0.002–0.11	0.06			

β = beta coefficient; 95% CI 95% confidence interval; HOMA-IR = homeostatic model assessment for IR; WH = waist-to-height-ratio, HbA1c = hemoglobin A 1c. * There were 354 participants available for analysis.

## Data Availability

The datasets used and/or analyzed during the current study are available from the corresponding author on reasonable request.

## Supplementary Materials

The following supporting information can be downloaded at: https://www.mdpi.com/article/10.3390/jcdd12050180/s1, Table S1: Demography, medication and measurements according to HOMA-IR tertiles for 358 participants; Table S2: Demography, medication and measurements according to glycemic status for 359 participants; Table S3: Uni- and multivariable linear regression models for 359 participants.
