# Supplementary material for: Left Ventricular Systolic Function in Asymptomatic Men Aged 65–75 Years, Relation to Insulin Resistance and Pre-Diabetes: A DANCAVAS Cross-Sectional Sub-Study
Source: J Cardiovasc Dev Dis. 2025 May 13;12(5):180. doi: 10.3390/jcdd12050180 (PMC12112607; doi:10.3390/jcdd12050180)
Supplement: Supplementary file 1 [file jcdd-12-00180-s001.zip › jcdd-3515209-supplementary.pdf]

Supplementary Table S1. Demography, medication and measurements according to HOMA-IR tertiles for 358 participants.

	**Lower HOMA-IR tertile (n = 119)**	**Middle HOMA-IR tertile (n = 124)**	**Higher HOMA-IR tertile (n = 115)**	**p - value**
Age, years	70 ± 3	70 ± 3	70 ± 3	0.95
Hypertension, n (%)	27 (23)	45 (36)	44 (38) *	**0.02**
Hypercholesterolemia, n (%)	35 (29)	37 (30)	48 (42)	0.08
Smoking, n (%)				
Active	18 (15)	11 (9)	10 (9)	0.20
Previous	55 (46)	67 (54)	63 (55)	0.32
Never	45 (38)	46 (36)	40 (35)	0.91
Pack-year	13.4 ± 16.7	15.0 ± 17.3	19.7 ± 21.9	0.15
Aspirin, n (%)	30 (25)	28 (23)	37 (32)	0.23
ACEIs/ARBs, n (%)	23 (19)	35 (28)	34 (30)	0.14
Beta-blocker, n (%)	3 (3)	8 (6)	11 (10)	0.07
Statins, n (%)	35 (29)	36 (29)	48 (42)	0.07
Duration statin treatment, months, median [IQR]	6 [3; 54]	13.5 [2; 73.5]	27 [4; 60]	0.57
Systolic blood pressure, mmHg	135 ± 18	138 ± 16	142 ± 18 *	**0.008**
Diastolic blood pressure, mmHg	78 ± 11	81 ± 10	82 ± 10 *	**0.009**
BMI, kg/m^2	25 ± 3	27 ± 3	29 ± 3 *	**< 0.001**
Waist circumference, cm	92 ± 9	100 ± 9	105 ± 9 *	**< 0.001**
Waist-to-height-ratio	0.51 ± 0.05	0.57 ± 0.05	0.59 ± 0.05 *	**< 0.001**
Hip, cm	100 ± 5	103 ± 6	106 ± 6 *	**< 0.001**
HbA1c, mmol/mol	35.6 ± 3.1	36.7 ± 3.6	38.0 ± 3.8 *	**< 0.001**
HDL, mmol/L	1.8 ± 0.4	1.5 ± 0.4	1.4 ± 0.4 *	**< 0.001**
LDL, mmol/L	2.8 ± 1.0	2.8 ± 1.0	2.7 ± 1.0	0.57
Total cholesterols, mmol/L	5.0 ± 1.1	4.9 ± 1.1	4.7 ± 1.0	0.23
Triglycerides, mmol/L	0.8 ± 0.3	1.1 ± 0.5	1.4 ± 0.7 *	**< 0.001**
Prediabetes, n (%)	24 (20)	58 (47)	82 (71) *	**< 0.001**
HOMA-IR, ** median [IQR]	1.4 [1.1; 1.7]	2.5 [2.2; 2.7]	4.5 [3.7;5.7] *	**< 0.001**
FPG, mmol/L	5.6 ± 0.4	5.9 ± 0.5	6.3 ± 0.7 *	**< 0.001**
2HPG, mmol/L	6.2 ± 1.6	6.8 ± 1.9	8.1 ± 2.4 *	**< 0.001**
hs-CRP, mg/L	1.8 ± 2.9	2.2 ± 2.4	2.4 ± 2.3 *	**< 0.001**

Continuous values are presented as mean ± standard deviation (SD) or median and [inter quartile range], and categorical data as number (n) and percentage (%). BMI = body mass index, ACEI = Angiotensin-converting enzyme inhibitors; ARB = Angiotensin receptor blockers; HbA1c = hemoglobin A 1c; HDL = high-density lipoprotein; LDL = low-density lipoprotein; HOMA-IR = homeostatatic model assessment for IR; FPG = fasting plasma glucose; 2HPG = two-hour plasma glucose; hs-CRP = high-sensitivity C-reactive protein.

*Higher vs. Lower HOMA-IR tertile, p < 0.05.

** HOMA-IR available for 358 participants

Supplementary Table S2. Demography, medication and measurements according to glycemic status for 359 participants.

	**Normal glucose tolerance (n = 194)**	**Pre-diabetes (n = 165)**	**p - value**
Age, years	70 ± 3	70 ± 3	0.33
Hypertension, n (%)	51 (26)	65 (39)	**0.008**
Hypercholesterolemia, n (%)	57 (29)	63 (38)	0.08
Smoking, n (%)			
Active	22 (11)	17 (10)	0.77
Previous	92 (47)	93 (56)	0.08
Never	79 (41)	53 (32)	0.10
Pack-years	12.2 ± 15.9	20.3 ± 21.0	**< 0.001**
Aspirin, n (%)	47 (24)	48 (29)	0.30
ACEIs/ARBs, n (%)	38 (20)	54 (33)	**0.004**
Beta-blocker, n (%)	8 (4)	14 (8)	0.09
Statins, n (%)	56 (29)	63 (38)	0.06
Duration statin treatment, months, median [IQR]	10 [2.5; 72]	18 [3; 52]	0.97
Systolic blood pressure, mmHg	135 ± 17	142 ± 18	**< 0.001**
Diastolic blood pressure, mmHg	79 ± 10	82 ± 11	**0.004**
BMI, kg/m^2	26 ± 3	28 ± 3	**< 0.001**
Waist circumference, cm	96 ± 10	103 ± 10	**< 0.001**
Waist-to-height-ratio	0.54 ± 0.05	0.58 ± 0.06	**< 0.001**
Hip, cm	102 ± 6	105 ± 6	**< 0.001**
HbA1c, mmol/mol	35.6 ± 3.2	38.2 ± 3.7	**< 0.001**
HDL, mmol/L	1.6 ± 0.4	1.5 ± 0.4	**0.02**
LDL, mmol/L	2.8 ± 1.0	2.8 ± 1.0	0.95
Total cholesterols, mmol/L	4.9 ± 1.0	4.8 ± 1.1	0.89
Triglycerides, mmol/L	1.0 ± 0.4	1.2 ± 0.7	**< 0.001**
HOMA 1,* median [IQR]	2.0 [1.4; 2.7]	3.1 [2.2; 4.9]	**< 0.001**
FPG, mmol/L	5.6 ± 0.3	6.3 ± 0.6	**< 0.001**
2HPG, mmol/L	5.9 ± 1.0	8.4 ± 2.4	**< 0.001**
hs-CRP, mg/L	1.9 ± 2.5	2.4 ± 2.6	**0.002**

Continuous values are presented as mean ± standard deviation (SD) or median and [inter quartile range], and categorical data as number (n) and percentage (%). BMI = body mass index; ACEI = Angiotensin-converting enzyme inhibitors; ARB = Angiotensin receptor blockers; BP = blood pressure; HbA1c = hemoglobin A 1c; HDL = high-density lipoprotein; LDL = low-density lipoprotein; HOMA-IR = homeostatic model assessment for IR; FPG = fasting plasma glucose; 2HPG = two-hour plasma glucose; hs-CRP = high-sensitivity C-reactive protein.

* HOMA-IR available for 358 participants

Supplementary Table S3. Uni- and multivariable linear regression models for 359 participants.

	**Univariable**			**Multivariable ***		
	**ß**	**95% CI**	**p - value**	**ß**	**95% CI**	**p - value**
**GLS**						
Pre-diabetes	0.16	-0.24-0.56	0.42			
TyG index	0.64	0.23-1.05	**0.002**	0.26	-0.18-0.70	0.25
Statin use	0.26	-0.16-0.68	0.23			
Hypertension	0.37	-0.06-0.79	0.09			
Systolic blood pressure, mmHg	0.01	0.003-0.03	**0.01**	0.01	-0.0005-0.02	0.05
Pack-years	-0.002	-0.01-0.008	0.67			
WH	7.9	4.6-11.3	**< 0.001**	6.7	3.1-10.3	**< 0.001**
Age	0.0008	-0.07-0.07	0.98			
HbA1c	0.05	-0.002-0.11	0.06			

β beta coefficient; 95% CI 95% confidence interval; TyG index = triglyceride glucose index; WH = waist-to-height-ratio, HbA1c = hemoglobin A 1c.

*There were 355 participants available for analysis
